# Supplementary material for: Amphotericin B Polymer Nanoparticles Show Efficacy against Candida Species Biofilms
Source: Pathogens. 2022 Jan 7;11(1):73. doi: 10.3390/pathogens11010073 (PMC8781556; doi:10.3390/pathogens11010073)
Supplement: Supplementary file 1 [file pathogens-11-00073-s001.zip › pathogens-1533852-supplementary.pptx]

## Slide 1
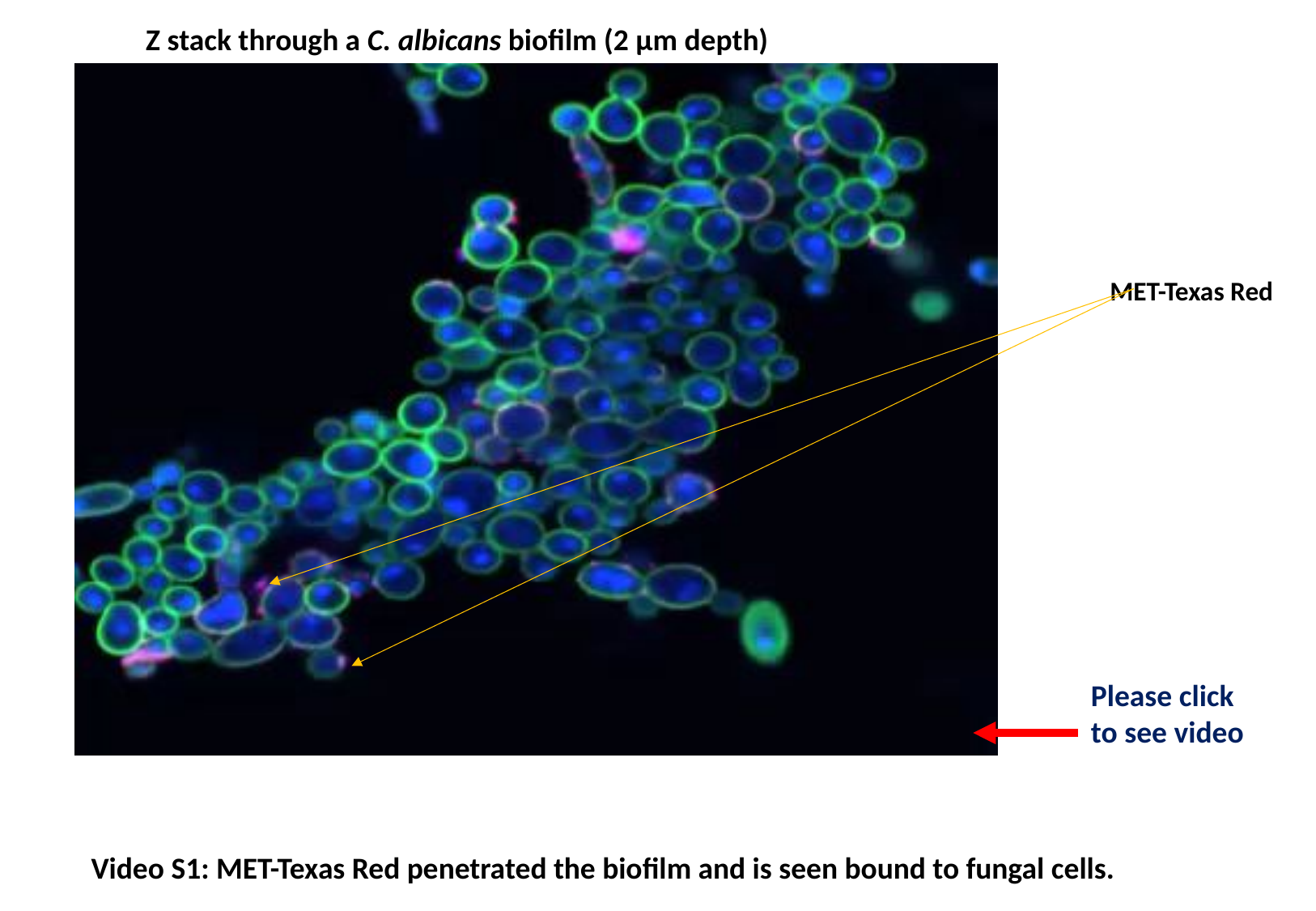

Z stack through a C. albicans biofilm (2 µm depth)
 MET-Texas Red
Please click to see video
Video S1: MET-Texas Red penetrated the biofilm and is seen bound to fungal cells.
